# Aberrant mRNA 5-methylcytosine modification contributes to ovarian dysfunction in a rat model of polycystic ovary syndrome

**DOI:** 10.1371/journal.pone.0358238

**Published:** 2026-10-27

**Authors:** Xiaohong Chen, Jing Lin, Zhoulin Chi, Sha Yang, Shuguang Yu

**Affiliations:** 1 Chengdu University of Traditional Chinese Medicine, Chengdu, China; 2 Guangxi University of Traditional Chinese Medicine, Nanning, China; 3 Key Laboratory of Acupuncture for Senile Disease (Chengdu University of TCM), Ministry of Education, Chengdu, China; 4 Acupoint Effects Key Laboratory of Sichuan Province, Chengdu, China; SNUH, KOREA, REPUBLIC OF

## Abstract

**Objective:**

This study investigated the altered landscape of messenger RNA 5-methylcytosine (m5C) methylation and the functional involvement of m5C-related regulators in a rat model of polycystic ovary syndrome (PCOS).

**Methods:**

A PCOS rat model was established by dehydroepiandrosterone (DHEA) induction, with the phenotype confirmed through estrous cycle monitoring, serum sex hormone analysis, and ovarian morphological assessment. Differentially m5C-methylated mRNAs were identified using an m5C epitranscriptomic microarray, and their associated biological pathways were predicted by KEGG analysis. Global m5C methylation levels and the expression and m5C enrichment of selected pathway-related genes were validated in ovarian tissues and isolated primary ovarian granulosa cells using dot blot, RT-qPCR, Western blotting, and methylated RNA immunoprecipitation quantitative PCR (MeRIP-qPCR). The functional roles of TET2 and NSUN6 were further evaluated using shRNA-mediated knockdown, followed by analysis of PI3K/AKT-related signaling proteins, serum sex hormones, and ovarian histopathology.

**Results:**

m5C modifications were predominantly localized within coding sequences and 3′ untranslated regions. Among 1,090 RNAs showing both differential m5C methylation and expression, 36 genes exhibited significant correlations between methylation and expression changes. Global mRNA m5C methylation was significantly elevated in the PCOS group. The m5C-related regulators Nsun6 and Tet2 showed increased m5C enrichment and significantly elevated protein expression. KEGG analysis linked the differentially modified transcripts to endocrine resistance and insulin-related signaling pathways. Tet2, Nsun6, Src, Pi3k, and Akt transcripts exhibited increased m5C enrichment in PCOS ovarian tissues and primary granulosa cells. Concurrently, SRC and PI3K protein expression was increased, whereas AKT protein expression was decreased in the PCOS group. Knockdown of TET2 and NSUN6 was confirmed at the mRNA and protein levels and differentially modulated PI3K/AKT-related protein expression and phosphorylation status, as assessed by PI3K, p-PI3K, AKT, and p-AKT levels. TET2 and NSUN6 knockdown also partially improved PCOS-associated abnormalities in serum FSH, LH, the LH/FSH ratio, testosterone, estradiol, and ovarian morphology.

**Conclusion:**

This study reveals a significantly altered mRNA m5C methylation landscape in a DHEA-induced PCOS rat model and primary ovarian granulosa cells. The functional knockdown data suggest that TET2 and NSUN6 may contribute to PCOS-associated ovarian dysfunction through differential regulation or remodeling of PI3K/AKT signaling. However, the opposite changes in the p-PI3K/PI3K and p-AKT/AKT ratios indicate that the pathway is differentially regulated rather than uniformly activated or inhibited. Because the DHEA model primarily mimics hyperandrogenism and MeRIP-qPCR does not provide single-nucleotide resolution, further validation in human samples and high-resolution m5C mapping is required to establish the precise molecular mechanism and clinical relevance of these findings.

## Introduction

Polycystic Ovary Syndrome (PCOS) typically emerges during puberty and ranks among the most common endocrine disorders affecting reproductive-aged women. Global epidemiological studies report PCOS prevalence rates of 8–13% in this population [1]. The disorder’s clinical hallmarks include oligo-ovulation or anovulation, hyperandrogenism, and insulin resistance, frequently coexisting with metabolic disturbances like obesity and dyslipidemia that increase susceptibility to cardiovascular disease and endometrial cancer [2]. While the exact etiology remains unclear, current evidence points to a complex interplay between environmental influences (including lifestyle factors and endocrine disruptors) and substantial genetic heterogeneity involving polygenic inheritance and epigenetic regulation [3,4].

Emerging research underscores the contribution of epigenetic dysregulation to PCOS pathophysiology, particularly through abnormal DNA methylation patterns, histone acetylation alterations, and non-coding RNA expression changes [5]. These modifications appear to disrupt inflammatory pathways, steroidogenic enzyme production, and insulin signaling, ultimately promoting metabolic dysfunction and endocrine imbalance [6]. Recent findings specifically implicate N6-methyladenosine (m6A) modification—a key epigenetic mechanism—in PCOS development [7,8]. Dysfunction of m6A regulatory proteins (“writers,” “erasers,” and “readers”) may drive disease progression, possibly by modulating chronic low-grade inflammation through inflammatory pathway activation [9,10]. However, the precise mechanistic relationships between DNA methylation and other epigenetic changes in PCOS require further elucidation.

5-Methylcytosine (m5C), a reversible RNA modification, critically regulates RNA stability, splicing, nuclear export, and translation [7]. This dynamic modification exerts widespread regulatory effects across diverse physiological and pathological processes. Emerging studies link m5C methylation to polycystic ovary syndrome (PCOS) through its modulation of metabolic and hormonal pathway genes [11,12]. The modification directly impacts insulin resistance, follicular dysfunction, and hyperandrogenism by altering the stability and translational efficiency of mRNAs governing glucose/lipid metabolism and steroidogenesis [11]. Key m5C regulators like YBX1 and ALYREF may further influence ovarian function and systemic metabolism through RNA-mediated mechanisms. The reversible nature of m5C positions it as a potential therapeutic target [13–15]. Investigating m5C distribution and function in ovarian, blood, and metabolic tissues could uncover new PCOS pathogenesis mechanisms and treatment strategies.

This study systematically analyzes m5C methylation profile alterations in a polycystic ovary syndrome (PCOS) rat model and investigates their functional impact on key signaling pathways, particularly those involving endocrine resistance and Hippo signaling. Endocrine resistance represents a prevalent metabolic abnormality in PCOS that strongly correlates with disease pathogenesis and progression. Our analysis of m5C methylation-gene expression relationships in PCOS rats reveals comprehensive methylation landscape changes, providing mechanistic insights for future investigations. Delineating these modifications’ functional consequences in critical pathways clarifies m5C methylation’s role in PCOS pathophysiology while potentially uncovering novel therapeutic targets. These findings advance both fundamental understanding of PCOS mechanisms and clinical translation for improved treatment strategies.

## Materials and methods

All animal experiments were approved by local ethical committee at Guangxi University of Chinese Medicine, People’s Republic of China (animal ethics approval number: DW20240603-145).

### Establishment of a rat model of PCOS

Six-week-old female Sprague-Dawley rats were purchased from Hunan Slaike Jingda Laboratory Animal Co., Ltd. and housed for one week under standard conditions (12:12 light-dark cycle, 24 ± 3°C, 45 ± 2% humidity). Vaginal smears collected daily at 8:00 AM tracked estrous cycle patterns. We randomly allocated ten rats with regular cycles to either control or PCOS groups. The PCOS group received 21 daily subcutaneous injections of 60 mg/kg dehydroepiandrosterone (DHEA) in 0.2 mL soybean oil, whereas controls received equivalent soybean oil volumes for 20 days [16].Five rats exhibiting persistent vaginal epithelial keratinization were randomly selected from each group (PCOS and control) and euthanized. We collected bilateral ovaries, randomly sampled them, embedded the samples in paraffin, sectioned them, and stained the sections with hematoxylin-eosin (HE) to assess ovarian morphology and verify PCOS induction. The remaining ovarian tissues were stored at −80°C for PCR and Western blot analysis. Ovaries from the other four rats per group were similarly preserved at −80°C for m5C meRIP-seq analysis [10].All rats were fasted for 12 hours with free access to water prior to experiments while conscious. Before sacrifice, anesthesia was induced and maintained using isoflurane inhalation (induction at 5% isoflurane, maintenance at approximately 2%, until the loss of righting reflex and pain reflex were confirmed). Subsequently, euthanasia was performed via cardiac exsanguination for blood sample collection, followed by cervical dislocation to ensure death. All anesthesia and euthanasia procedures were performed by trained personnel in accordance with the ARRIVE guidelines and AVMA recommendations to minimize animal suffering (Ethical Approval: DW20240603−145, Institution: Guangxi University of Chinese Medicine).

### Hematoxylin-eosin (HE) staining

The ovarian tissues were fixed with 4% paraformaldehyde, dehydrated through an ethanol gradient, and embedded in paraffin. Sections of 4 μm thickness were prepared using a microtome. After dewaxing and rehydration, the sections were stained with hematoxylin for 15 minutes to visualize nuclei, differentiated with 1% acid ethanol, and counterstained with 1% eosin to highlight cytoplasmic structures. Finally, the sections were dehydrated, cleared, and mounted with neutral resin.Both the experimental and Blank groups of ovarian tissues were processed following this hematoxylin-eosin (HE) staining protocol. The number and morphological changes of primordial follicles, early antral follicles, atretic follicles, and corpora lutea were observed under an optical microscope, serving as morphological criteria to evaluate the successful establishment of the polycystic ovary syndrome (PCOS) model [17].

### RNA extraction and quantification

Total RNA was extracted from ovarian tissues using TRIzol reagent (Thermo Scientific #15596026) according to the manufacturer’s protocol. Residual genomic DNA was subsequently removed by DNase I treatment (NEB #M0303L). RNA purity was assessed by measuring the A260/A280 ratio using a Nanodrop OneC spectrophotometer, while RNA integrity was evaluated using the LabChip GX Touch system (Revvity). RNA concentration was quantified using a Qubit 3.0 fluorometer with the Qubit RNA Broad Range Assay kit (Thermo Scientific #Q10210).

### Enrichment of Polyadenylated RNA (polyA + RNA)

Polyadenylated RNA was enriched from total RNA using VAHTS mRNA Capture Beads (Vazyme, China, cat. #N401) according to the manufacturer’s protocol. This isolation step specifically targets polyA + RNA molecules, enabling downstream mRNA analysis.

### m5C meRIP experiment

For the m5C methylated RNA immunoprecipitation (meRIP) assay, polyA + RNA was treated with 20 mM ZnCl_2_ at 95°C for 5–10 minutes, with 10% retained as input control. The remaining RNA was incubated with anti-m5C antibody (Diagenode #C15200081) and RNasin (Promega #N2615, 40 U/μL) under rotation at 4°C for 2 hours. Protein A magnetic beads (Thermo #88848) were then added and incubated for 1 hour to capture RNA-antibody complexes. After washing, immunoprecipitated RNA was extracted using TRIzol reagent.

### m5C meRIP library preparation and sequencing

For library preparation, both input and immunoprecipitated RNA were processed using the KCTM Digital mRNA Library Prep Kit (Seqhealth Tech. Co., Ltd., Wuhan, China) following the manufacturer’s protocol. The kit employs 12-base unique molecular identifiers (UIDs) to tag cDNA molecules, thereby minimizing PCR duplicates and sequencing artifacts. PCR products spanning 200–500 base pairs were size-selected during library construction. Quantified libraries underwent paired-end sequencing (PE150) on an Illumina NovaSeq X Plus platform. The raw fastq data from this transcriptome study are available in the Genome Sequence Archive under accession number CRA026876 (https://ngdc.cncb.ac.cn/gsa/s/d7e9Yd83). Seqhealth Technology Co., Ltd (Wuhan, China) performed all m5C meRIP-seq procedures, including library preparation, sequencing, and data analysis [18].

### In vivo TET2 and NSUN6 knockdown

A total of 30 Sprague–Dawley rats were randomly assigned to five groups, with six rats in each group (n = 6): the control group, the DHEA-induced PCOS model group, the PCOS+sh-NC group, the PCOS+sh-TET2 group, and the PCOS+sh-NSUN6 group. The PCOS model was established using a previously published DHEA-induced protocol [16].

Rats scheduled for ovarian lentiviral injection were fasted for 12 h before surgery. Anesthesia was induced with 2% isoflurane at a fresh gas flow rate of 4 L/min and a delivery rate of 0.41 mL/min. Each rat was placed in lateral recumbency, and a 1–2 cm abdominal incision was made bilaterally. The skin, fascia, and muscle layers were carefully separated to expose the ovaries. Using a 50-μL microsyringe, sh-TET2, sh-NSUN6, or sh-NC lentiviral vectors (Shanghai Jikang Gene Pharmaceutical Co., Ltd.; titer, 5 × 10^^8^ TU/mL) were slowly injected into the ovarian tissue. The needle was left in place for 5 min after each injection to minimize leakage. A total volume of 30 μL was administered to each ovary and divided between two injection sites, resulting in a total injection volume of 60 μL per rat. The control and PCOS model groups received the corresponding vehicle treatment.

Penicillin was administered intramuscularly at 80,000 U/kg according to the approved animal protocol. The penicillin solution was prepared at 80,000 U/mL, and the injection volume was calculated according to the body weight of each rat. To verify lentiviral transduction efficiency and localization in ovarian granulosa cells, a separate subset of rats was injected with Lenti-GFP, and GFP fluorescence was examined in frozen ovarian sections. At the end of the experimental period, ovarian tissues were collected to assess TET2 and NSUN6 knockdown efficiency by RT-qPCR and Western blotting. PI3K/AKT-related proteins, serum FSH, LH, LH/FSH, testosterone, estradiol, and ovarian histopathology were subsequently evaluated.

### RT-qPCR

Total RNA was extracted from tissues or cells using Trizol reagent (Takara, 9109). cDNA was synthesized using HiScript III All-in-one RT SuperMix Perfect for qPCR (Vazyme, R333-01). cDNA was detected using Sybr-green real-time fluorescence quantitative PCR. Gene expression was detected using Sybr-green real-time fluorescence quantitative PCR. (ChamQ Universal SYBR qPCR Master Mix, Vazyme, Q711-02) The qPCR primer sequences are shown in S1 Table. mRNA expression levels were normalized to β-actin expression levels.

### Western-blot

Western blotting was performed as previously described. Briefly, we generated total protein from each group by sodium dodecyl sulfate (SDS)-polyacrylamide gel electrophoresis and transferred the proteins to polyvinylidene fluoride membranes. The membranes were incubated with the primary antibody PI3K (1:1000; ABclonal,Cat. No. A11177,), AKT(1:2000; Proteintech,Cat. No.10176-2-AP), Src (c-SRC,1:3000; Proteintech Cat. No.11097-1-AP),TET2 (Abcam, Cat. No. ab94580, 1:1000), NSUN6 (Abcam, Cat. No. ab197342, 1:1000), and GAPDH(1:5000; Abcam,Cat. No. ab8245).overnight at 4 °Cand then incubated with secondary antibodies specific to the mouse-, rabbit-primary antibodies (1:4,000 Santa Cruz Biotechnology) at room temperature for 1 h. Afterward, the membranes were exposed using an enhanced chemiluminescence reagent (Chemicon International, USA). Full-length original western blots in this article are provided in Supplementary File.

### Gene-specific MeRIP-qPCR

The analysis of m5C modifications in specific genes was performed using the GenSeq® m5C MeRIP Kit (Shanghai Yunce Biotechnology Co., Ltd., GS-ET-003) according to the manufacturer’s instructions [10,19]. Briefly, 100 μg of total RNA was fragmented to approximately 100-nucleotide-long segments using metal ion-induced hydrolysis. Prior to immunoprecipitation, one-tenth of the fragmented RNA was retained as an input control. Subsequently, input, m5C, and IgG groups were prepared for size determination. The RNA samples were then incubated overnight at 4°C with either an m5C antibody (anti-5-methylcytosine antibody, 1:200 dilution, Abcam) or IgG (mouse IgG1 isotype control antibody, 1:5000 dilution, Abcam). Antibody-RNA complexes were captured using protein A/G magnetic beads and thoroughly washed to remove nonspecifically bound material. After elution, the RNA was purified using the RNeasy Mini Kit (QIAGEN). Both input RNA and immunoprecipitated m5C-modified RNA were reverse-transcribed using the PrimeScript RT Reagent Kit (Takara). The levels of target transcripts were determined by quantitative PCR analysis, and the enrichment of m5C modifications was calculated relative to the input group. The primer sequences for MeRIP-qPCR are listed in S2 Table.

### Statistical analysis

Differentially methylated and/or expressed mRNAs were identified using a fold-change threshold of ≥1.5 and a nominal *P* value <0.05. Multiple testing was controlled using the Benjamini–Hochberg method, and false discovery rates (FDRs) were reported. Because the microarray analysis included three biological replicates per group (*n* = 3), these findings were considered exploratory and were further validated by RT-qPCR, MeRIP-qPCR, and Western blotting. For the validation experiments, six independent biological replicates were included per group (*n* = 6). Technical replicates were averaged before statistical analysis and were not treated as independent samples. Data were analyzed using GraphPad Prism 8.0 and SPSS version 26.0 and are presented as mean±SD. Two-group comparisons were performed using an unpaired two-tailed Student’s *t*-*t*est or Mann–Whitney *U* test, as appropriate. Comparisons involving more than two groups were performed using one-way ANOVA followed by Tukey’s or Dunnett’s multiple-comparison test, or the Kruskal–Wallis test followed by Dunn’s test. A two-sided *P* < 0.05 was considered statistically significant.

## Results

### Establishment of the PCOS rat model

To further investigate the m5C gene modification patterns in polycystic ovary syndrome (PCOS), a dehydroepiandrosterone (DHEA)-induced rat model of PCOS was established [17]. Two weeks after DHEA administration, a significant difference in body weight was observed between the PCOS group and the Blank group. By day 21, the body weight of PCOS rats (Fig 1A) was significantly higher than that of the Blank group, while ovarian weight in the PCOS group also showed a marked increase (Fig 1B). Serum analysis revealed significantly decreased FSH and E2 levels in PCOS rats (Fig 1C and 1G), accompanied by elevated LH levels, an increased LH/FSH ratio, and higher testosterone concentrations (Fig 1D–1F). Vaginal smears collected from the second week onward demonstrated disrupted estrous cycles with irregular phases in the PCOS group (Fig 1H). Histological examination of H&E-stained ovarian sections showed that PCOS rats exhibited typical polycystic ovarian morphology with thickened theca interna cells compared to the Blank group (Fig 1I and 1J). These findings are consistent with the diagnostic criteria for polycystic ovary syndrome.

**Fig 1:**
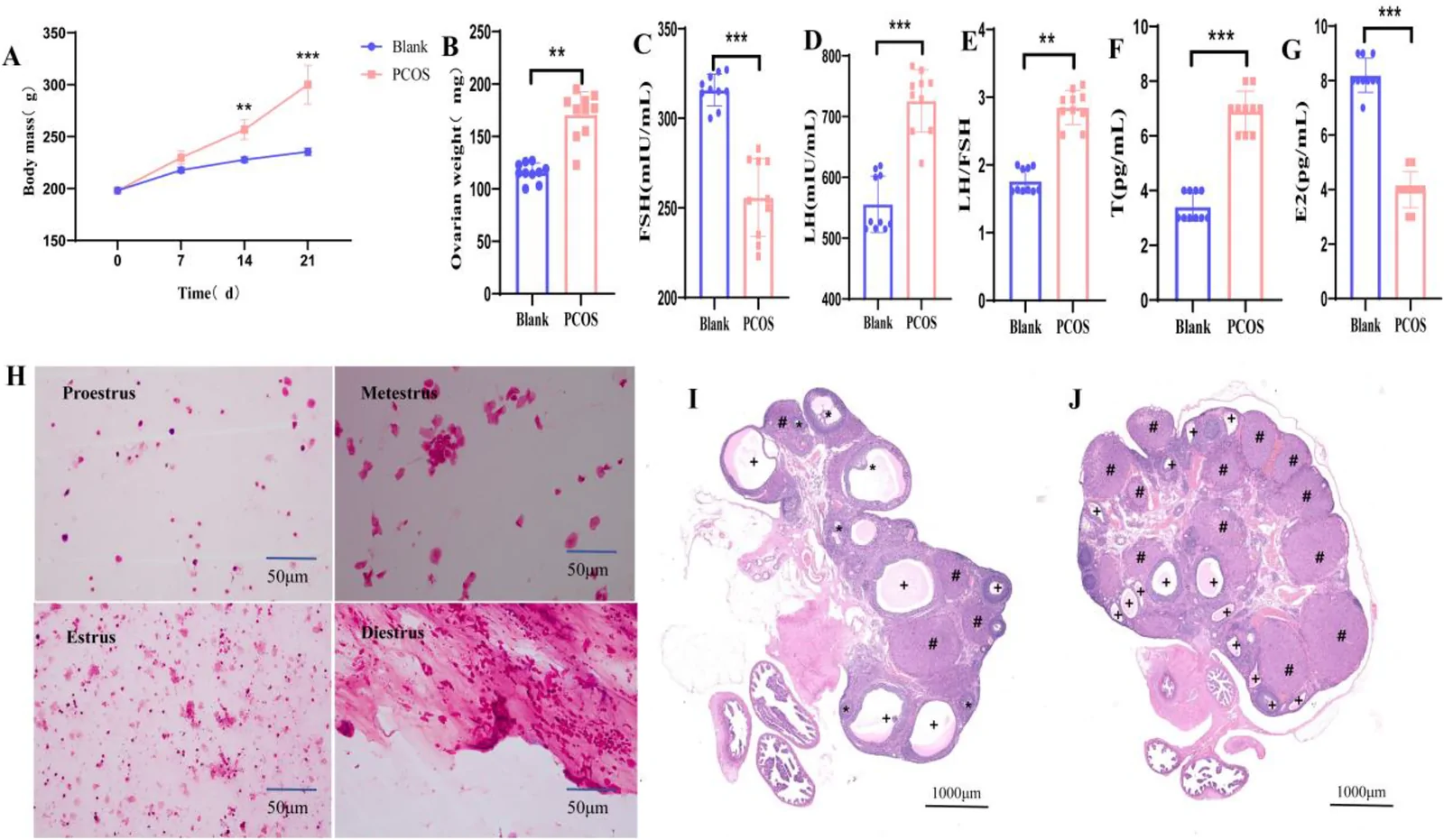
Characteristics of the polycystic ovary syndrome rat model. (https://doi.org/10.6084/m9.figshare.334154080). (A) The PCOS model group showed significantly higher body weight than Blank rats by day 21 after DHEA administration, with differences emerging from day 14 (n = 10 per group). (B) Ovarian weight increased significantly in PCOS rats compared to Blank rats by day 21 (n = 6 per group). (C) PCOS rats exhibited significantly lower FSH levels than Blanks (n = 10 per group). (D) LH levels were markedly higher in the PCOS group (n = 10 per group). (E) The LH/FSH ratio rose significantly in PCOS animals (n = 10per group). (F) Testosterone concentrations were substantially elevated in the PCOS group (n = 10 per group). (G) E2 levels decreased significantly in PCOS rats (n = 10 per group). (H) From week 3 onward, PCOS rats displayed irregular estrous cycles, unlike the regular cycles in Blanks (n = 10 per group). (I, J) H&E-stained ovarian sections from PCOS rats showed characteristic polycystic changes with thickened follicular membranes at 100 × magnification (n = 6 per group). *: developing follicles; ^#^: corpus luteum; ^+^: cystic follicles.* *P* < 0.05, ** *P* < 0.01, *** *P* < 0.001. PCOS, polycystic ovary syndrome.

Two weeks after DHEA administration, body weight differed significantly between the PCOS and Blank group rats. By day 21, PCOS rats exhibited greater body weight (Fig 1A) and ovarian weight (Fig 1B) compared to the Blank group. Serum analysis revealed significantly lower FSH and E2 levels in the PCOS group (Fig 1C and 1G), alongside elevated LH levels, an increased LH/FSH ratio, and higher testosterone concentrations (Fig 1D–1F). Vaginal smears collected from week 2 onward demonstrated disrupted estrous cycles in PCOS rats (Fig 1H). H&E-stained ovarian sections showed that PCOS ovaries displayed characteristic polycystic morphology with thickened follicular endometrial cells, contrasting with Blank group specimens (Fig 1I and 1J). These observations align with established polycystic ovary syndrome diagnostic criteria.

### Transcriptome-wide m^5^C methylation of mRNA in the ovaries of the blank and PCOS groups

m^5^C meRIP-seq analysis of rat ovaries identified 9,676 m^5^C peaks in the Blank group and 10,706 peaks in the PCOS group, with 8,252 peaks shared between both groups (Fig 2A and 2B). Among 12,130 genes exhibiting m^5^C modifications in both groups, 2,454 showed methylation exclusively in Blanks, while 1,424 were uniquely methylated in PCOS samples (Fig 2A and 2B). Peak-annotated genes displayed region-specific methylation patterns, visualized through distribution plots and heatmaps (Fig 2C and 2D). Transcripts typically contained 1–3 m^5^C peaks, with pronounced enrichment near CDS and 3′UTR regions (Fig 2E and 2F). In Blanks, 61.56% of peaks localized to CDS, 35.47% to 3′UTR, 2.86% to 5′UTR, and 0.11% to npExons (Fig 2E). PCOS samples showed comparable distribution, with 64.82% in CDS, 32.52% in 3′UTR, 2.57% in 5′UTR, and 0.09% in npExons (Fig 2F). Five significant sequence motifs emerged from m^5^C-modified regions, revealing distinct deposition patterns between normal and PCOS tissues (Fig 2G and 2H). m5C-MeRIP-seq identified 1,090 differentially methylated genes (DMGs, fold change ≥1.5, *P* < 0.05), with 735 showing elevated and 355 showing reduced methylation (Fig 3A). The ten most markedly altered m5C-modified mRNAs included Itgax, Pla2r1, Cep162, and 17 additional genes (Table 1).

**Table 1:** Significantly differentially methylated mRNAs.

GeneName	Regulation	Lg2FCD	RNA length	*P* value	FDR
Itgax	hyper	9.885338696632212	62	0.0215556707205909	0.992408932605865
Pla2r1	hyper	9.885338696632212	152	0.0340748243155014	0.992408932605865
Cep162	hyper	9.885338696632212	182	0.0309739563642094	0.992408932605865
Mark1	hyper	9.885338696632212	182	0.0228382831565368	0.992408932605865
Cpne5	hyper	9.885338696632212	1379	0.0361296342049441	0.992408932605865
Ncs1	hypo	−9.174457808	62	0.0156814544610463	0.992408932605865
Magee1	hypo	−9.174457808	62	0.0189570549831282	0.992408932605865
Rae1	hypo	−9.022454715	209	0.035722324203131	0.992408932605865
Ppp2r1b	hypo	−9.022454715	62	0.0427587584786792	0.992408932605865
Bco1	hypo	−8.852529713	91	0.0266251555086242	0.992408932605865

**Fig 2:**
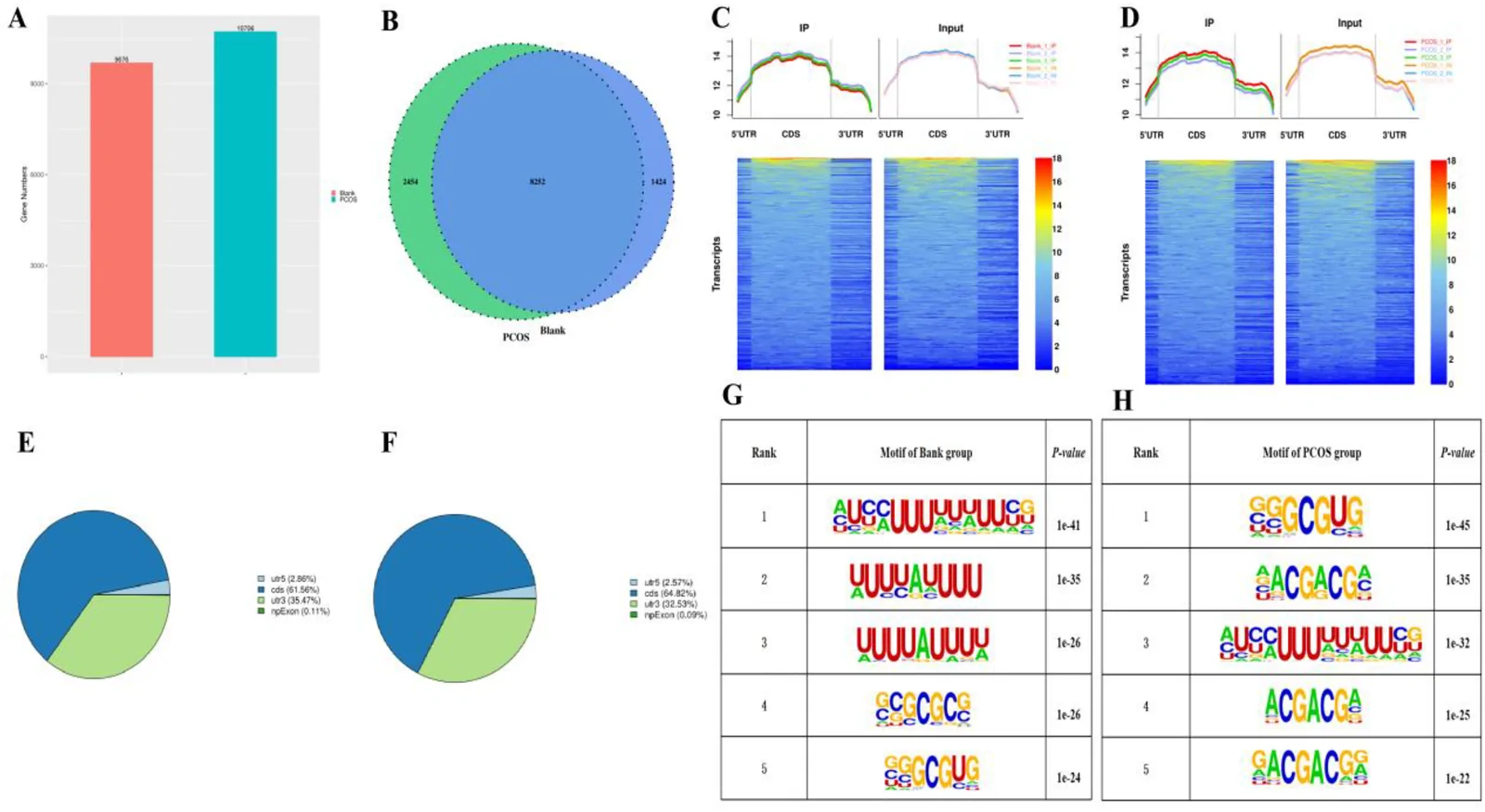
Overview of m5C gene modifications in the ovaries of polycystic ovary syndrome (PCOS) model rats. (https://doi.org/10.6084/m9.figshare.33415426). (A) The ovaries of control (Blank) and PCOS groups exhibited distinct numbers of m5C-modified genes. (B) A Venn diagram depicts the overlap in m5C-modified genes between Blank and PCOS groups. (C, D) Heatmaps display the distribution of sequencing reads across peak-associated functional regions in the Blank (C) and PCOS (D) groups. (E, F) Pie charts illustrate the genomic distribution of m5C peaks in the Blank (E) and PCOS (F) groups. (G, H) The top five enriched sequence motifs linked to m5C peaks are shown for the Blank (G) and PCOS (H) groups. N = 3.

**Fig 3:**
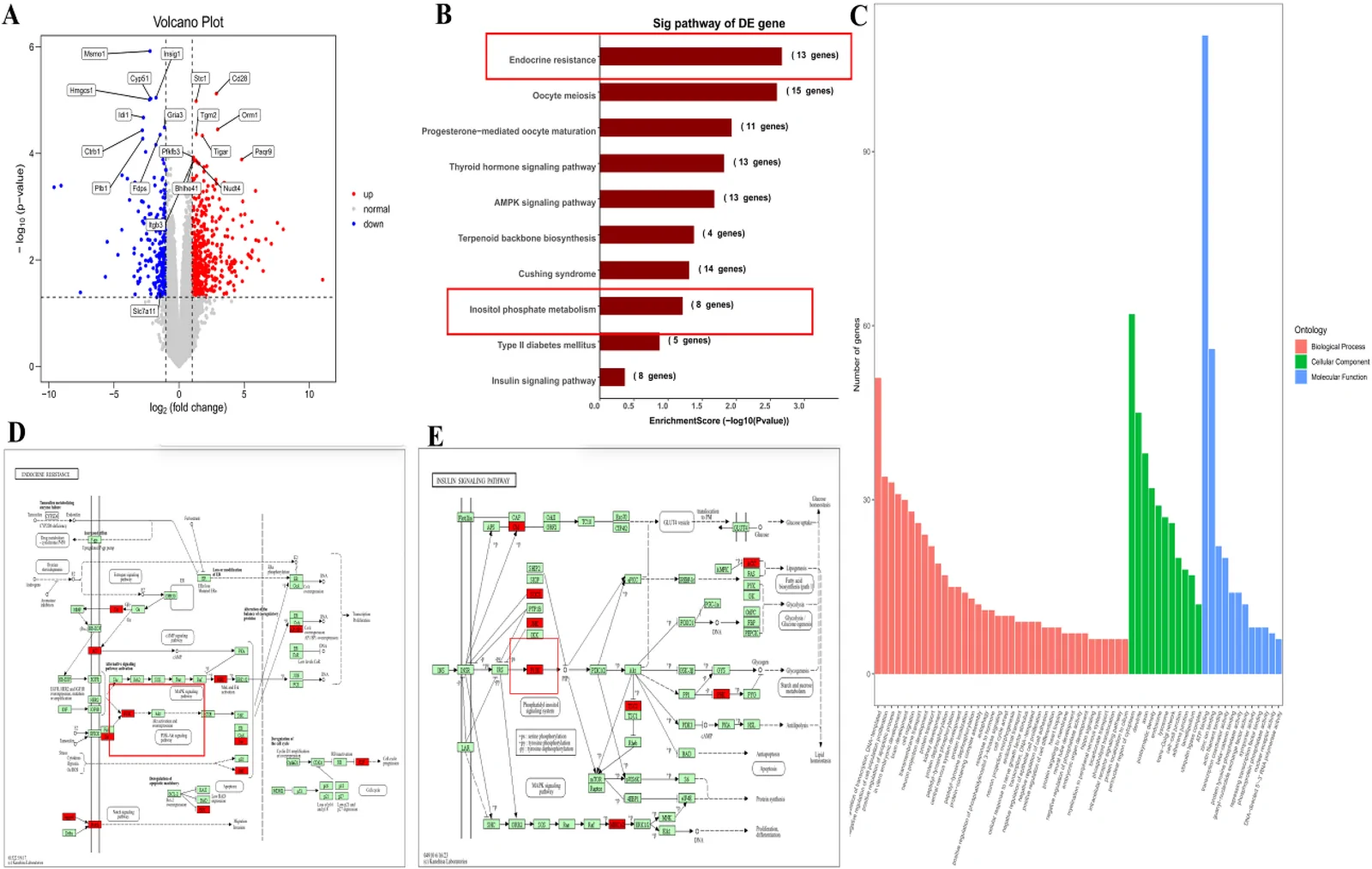
Analysis of differentially m5C-modified genes between Blank and PCOS groups. (https://doi.org/10.6084/m9.figshare.33415453). (A) Volcano plot displaying differentially methylated m5C peaks between the Blank group and the polycystic ovary syndrome (PCOS) group. (B) Kyoto Encyclopedia of Genes and Genomes (KEGG) analysis revealing the top 10 significantly enriched pathways.(C) Gene Ontology (GO) enrichment analysis of differentially methylated m5C genes in the two groups. (D) KEGG pathway enrichment map highlighting the endocrine resistance signaling pathway. (E) KEGG pathway enrichment map highlighting the Insulin signaling pathway. Differentially expressed mRNAs with altered m5C methylation levels are marked in red. Pathway diagrams were adapted from the KEGG and Genome Pathway databases. N = 3.

Furthermore, we observed an upregulation of m^5^C-modifying “writer” (methyltransferase) and “eraser” (demethylase) enzymes in the PCOS group, including NOP2/Sun RNA Methyltransferase 6 (NSUN6) (Fold change = 3.98107170553497, *P* < 0.001) and Ten-Eleven Translocation 2 (TET2) (Fold change = 1.16175646936305, *P* < 0.001).

### Functional analysis of differentially methylated transcripts in the two groups

Comparative methylation analysis identified 1,090 differentially methylated m^5^C peaks between the Blank group and the PCOS group, comprising 762 hypermethylated peaks and 328 hypomethylated peaks. The top 10 hypermethylated and hypomethylated genes are labeled on the volcano plot (Fig 3A). Kyoto Encyclopedia of Genes and Genomes (KEGG) pathway enrichment analysis revealed that the differentially methylated mRNAs were involved in 22 significantly enriched pathways. The top 10 pathways with the highest enrichment scores are shown in Fig 3B, including endocrine resistance, oocyte meiosis, progesterone-mediated oocyte maturation, thyroid hormone signaling pathway, AMPK signaling pathway, terpenoid backbone biosynthesis, Cushing syndrome, inositol phosphate metabolism, type II diabetes mellitus, and insulin signaling pathway. Among these, the endocrine resistance pathway was the most significantly enriched (containing 13 differentially methylated mRNAs), followed by the oocyte meiosis pathway (containing 15 mRNAs) (Fig 3B). Gene Ontology (GO) enrichment analysis indicated that within biological processes (BP), “positive regulation of DNA methylation-dependent heterochromatin assembly” obtained the highest enrichment score; within cellular components (CC), “perinuclear region of cytoplasm” was the most enriched; and within molecular functions (MF), “ATP binding” received the highest m^5^C methylation enrichment score (Fig 3C).

To validate the methylation level of a key gene involved in endocrine resistance, Src proto-oncogene, non-receptor tyrosine kinase (SRC), was selected for verification. MeRIP-PCR results confirmed that SRC mRNA indeed exhibited a higher methylation level (P < 0.05) (Fig 4A). Furthermore, prediction of SRC mRNA using the iRNA-m^5^C program identified four potential m^5^C sites with high confidence (S2 Table).

**Fig 4:**
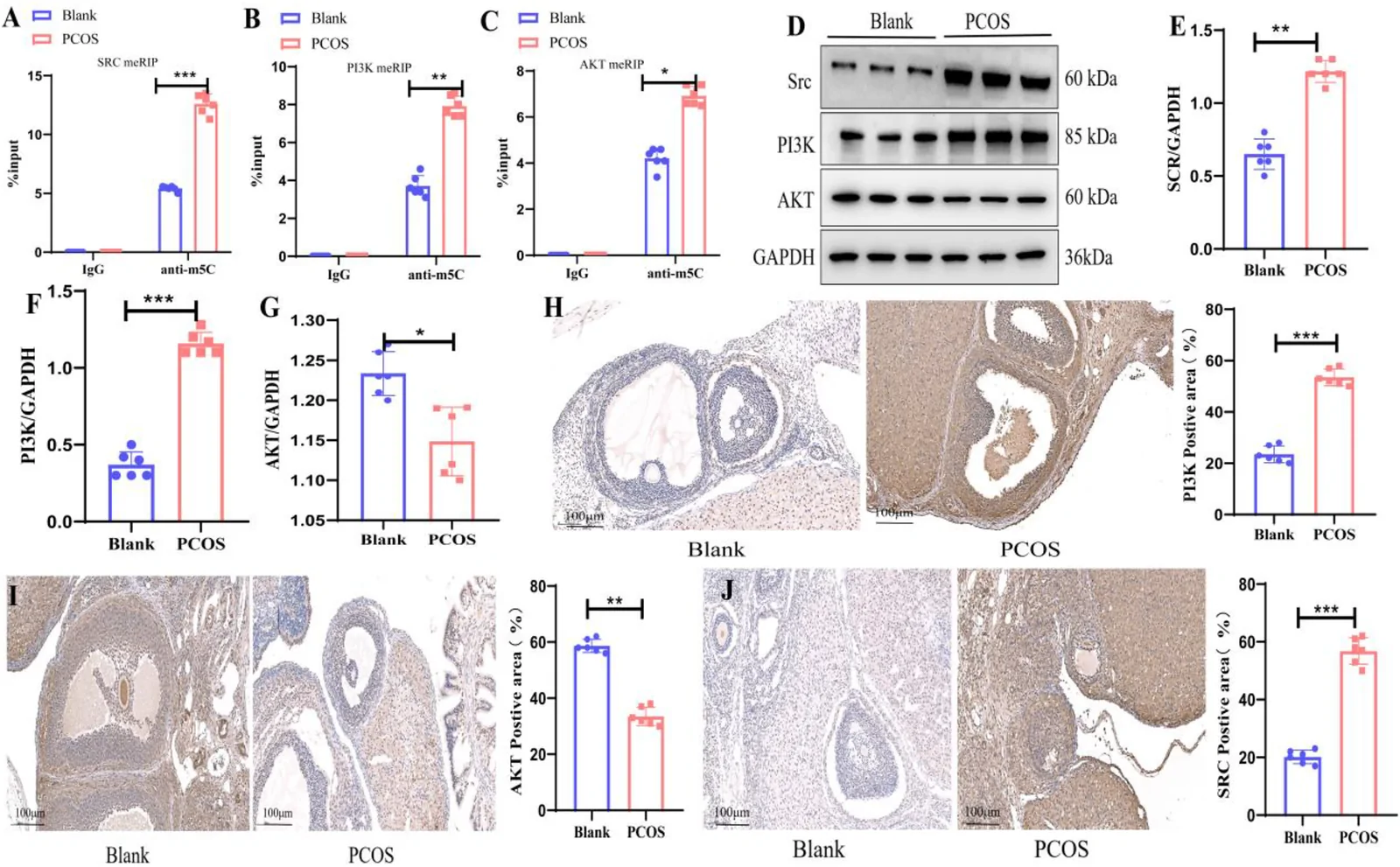
Inhibition of the Phosphatidylinositol 3-Kinase (PI3K)/Protein Kinase B (AKT) signaling pathway. (https://doi.org/10.6084/m9.figshare.33415468). (A-C) The m^5^C modification levels of SRC (A), PI3K (B), and AKT (C) mRNA in different samples were measured using the MeRIP assay.(D) Representative images of Western blot analysis detecting SRC, PI3K, and AKT protein expression in the Blank and PCOS groups. (E–G) Bar graphs showing the quantitative comparison of SRC, PI3K, and AKT protein expression levels.(H–J) Representative immunohistochemical images and corresponding statistical analyses of PI3K, AKT, and SRC protein expression. Data are presented as mean ± standard deviation; N = 6. Compared with the Blank group, *P < 0.05, **P < 0.01, ***P < 0.001.

### Potential involvement of m5C modification in the dysregulation of the SRC/PI3K/AKT pathway

Kyoto Encyclopedia of Genes and Genomes (KEGG) pathway enrichment analysis of endocrine resistance and insulin signaling pathways associated with the polycystic ovary syndrome (PCOS) phenotype implicated methylation modifications of the PI3K/AKT pathway in PCOS (Fig 3D and 3E). To identify potential upstream regulators underlying this altered methylation landscape, we performed a global mRNA m5C quantitative analysis of key m5C regulatory enzymes. The m5C methylation levels of the m5C methyltransferase NSUN6 and the modifier enzyme TET2 were significantly higher in the PCOS group compared to the Blank group (Fig 4A and 4B). Consistent with the upregulation of m5C methylation levels of these m5C regulatory factors, the mRNA levels of the m5C methyltransferase *NSUN6* and the modifier enzyme *TET2* were elevated (Fig 4F and 4G). Furthermore, Western blot (WB) and results demonstrated that their protein expression was significantly upregulated in the PCOS group(Fig 4K a-b, 4L and 4M).We then employed methylated RNA immunoprecipitation quantitative polymerase chain reaction (MeRIP-qPCR), Western blot, and immunohistochemistry to assess Scr/PI3K/AKT signaling pathway activity within the endocrine resistance and insulin signaling pathways. MeRIP-qPCR revealed hypermethylation of the Scr, PI3K and AKT genes in PCOS model rats (Fig 4C–4E). The mRNA levels of the *Scr*, *PI3K* and *AKT* mRNA were elevated(Fig 4H–4J).Western blot and immunohistochemistry demonstrated that, compared with the Blank group, AKT protein expression was significantly decreased, while PI3K protein expression was significantly increased in the PCOS group (Fig 4K c-e and 4N–4P). The expression of SRC (Src family tyrosine kinase), an upstream regulator of the PI3K-AKT pathway [20], was also significantly upregulated in the PCOS group (Fig 4K c-e and 4N–4P)). Together, these findings suggest that the upregulation of m5C regulatory enzymes, including NSUN6 and TET2, is closely associated with hypermethylation of the SRC/PI3K/AKT axis. This aberrant methylation pattern may contribute to the overactivation of PI3K and impaired AKT function, consequently promoting endocrine resistance and abnormal insulin signaling.

### Validation of the m5C regulators and PI3K/AKT pathway in primary ovarian granulosa cells

Given the high heterogeneity of ovarian tissue, which comprises multiple cell types, we aimed to investigate whether the observed molecular alterations in whole ovarian tissue specifically correlate with granulosa cells [21,22]. These cells are pivotal in follicular development and the pathogenesis of polycystic ovary syndrome (PCOS). To address this, primary ovarian granulosa cells (PGCs) were isolated and purified from both PCOS model rats and control (Blank) rats. Total RNA was extracted, and global RNA m5C methylation levels were evaluated via dot blot analysis. The results demonstrated a significant increase in the overall m5C methylation level in PGCs from PCOS rats compared to the Blank group (Fig 5A).

**Fig 5:**
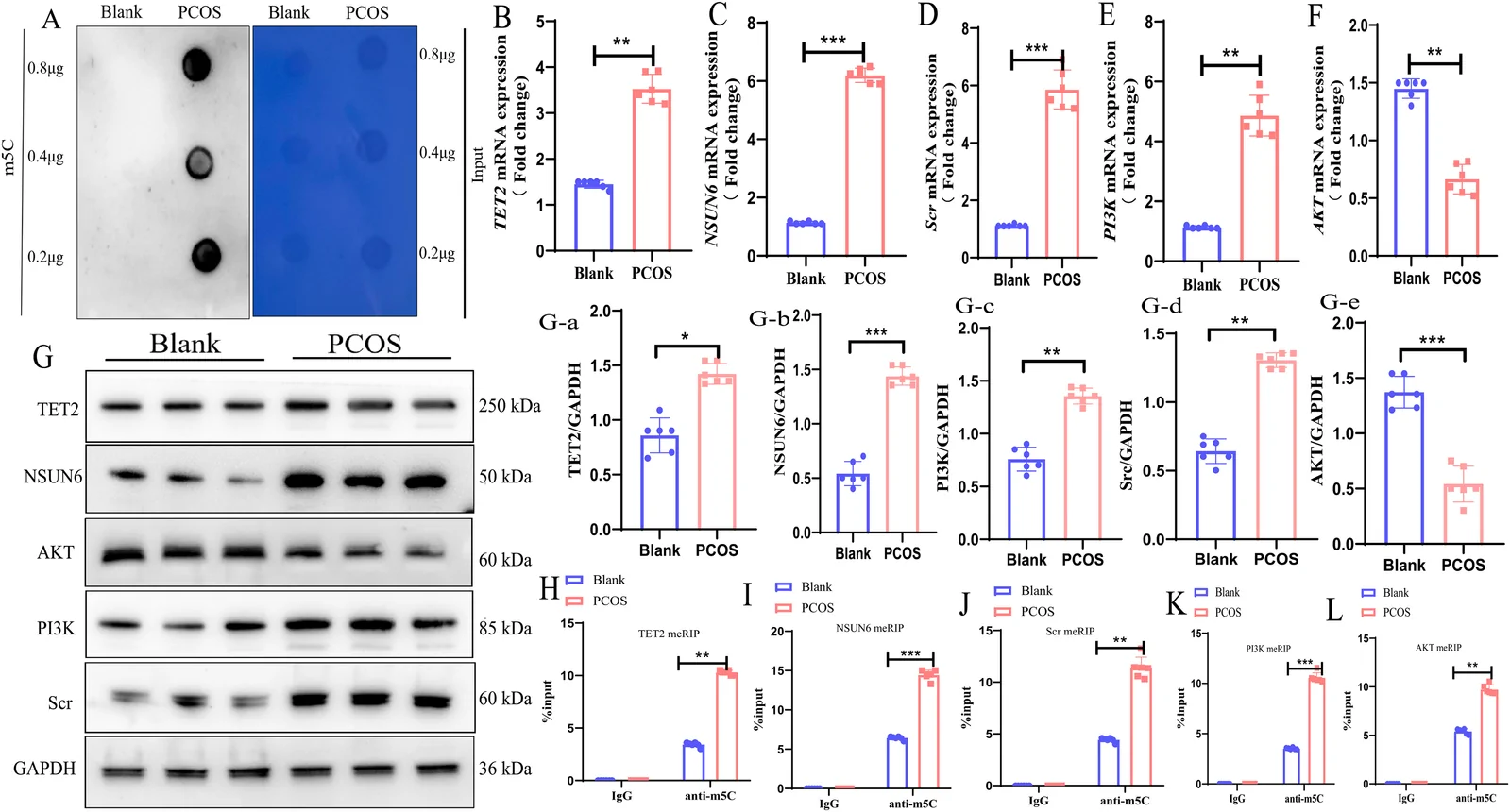
Validation of the m5C regulators and SRC/PI3K/AKT pathway in primary ovarian granulosa cells. (https://doi.org/10.6084/m9.figshare.31440736). (A) Dot blot analysis and corresponding quantification of global mRNA m5C methylation levels in PGCs isolated from the Blank and PCOS rat groups. Methylene blue staining was used as a loading control. (B-F) The relative mRNA expression levels of Nsun6 (B), Tet2 (C), Src (D), Pi3k (E), and Akt (F) in PGCs were determined by RT-qPCR. (G) Representative Western blot images and quantitative analysis of NSUN6, TET2, SRC, PI3K, and AKT protein levels in PGCs (a-e represents the quantification of each respective protein). GAPDH was used as the internal control. (H-L) MeRIP-qPCR analysis was performed to assess the relative m5C methylation enrichment of Tet2 (H), Nsun6 (I), Src (J), Pi3k (K), and Akt (L) mRNAs in PGCs from the Blank and PCOS groups. Data are presented as the mean ± SD (or SEM). n = 6 per group. *P < 0.05, **P < 0.01, ***P < 0.001.vs. Blank group.

Subsequent RT-qPCR and Western blot analyses indicated that the expression levels of the m5C methyltransferase NSUN6 and the modifier TET2 were significantly elevated in PCOS-derived PGCs relative to the control group (Fig 5B, 5C and 5G a–b). Concurrently, the expression levels of SRC and PI3K were markedly upregulated, while both the mRNA and protein levels of AKT were significantly downregulated in these cells (Fig 5D–5F and 5G c–e). Furthermore, MeRIP-qPCR analysis revealed a hypermethylation status of TET2, NSUN6, SRC, PI3K, and AKT mRNAs in PGCs from the PCOS group (Fig 5H–5L).These findings at the primary cell level are consistent with our in vivo whole-ovary data. Together, they suggest a potential link between the dysregulation of m5C regulatory enzymes and alterations in the SRC/PI3K/AKT signaling pathway, underscoring these changes as distinct molecular characteristics of granulosa cells within the PCOS microenvironment.

### TET2 and NSUN6 knockdown modulates PI3K/AKT signaling and PCOS-associated ovarian abnormalities in DHEA-induced rats

To investigate the functional roles of TET2 and NSUN6 in PCOS, we performed gene knockdown experiments in the DHEA-induced PCOS rat model. As shown in Fig 6A–6C, TET2 knockdown efficiency was confirmed by qRT-PCR and Western blotting. Compared with the corresponding negative-control group, TET2-specific siRNA significantly reduced TET2 mRNA and protein expression. Similarly, NSUN6-specific siRNA significantly reduced NSUN6 mRNA and protein levels, confirming effective NSUN6 knockdown (Fig 6D–6F).

**Fig 6:**
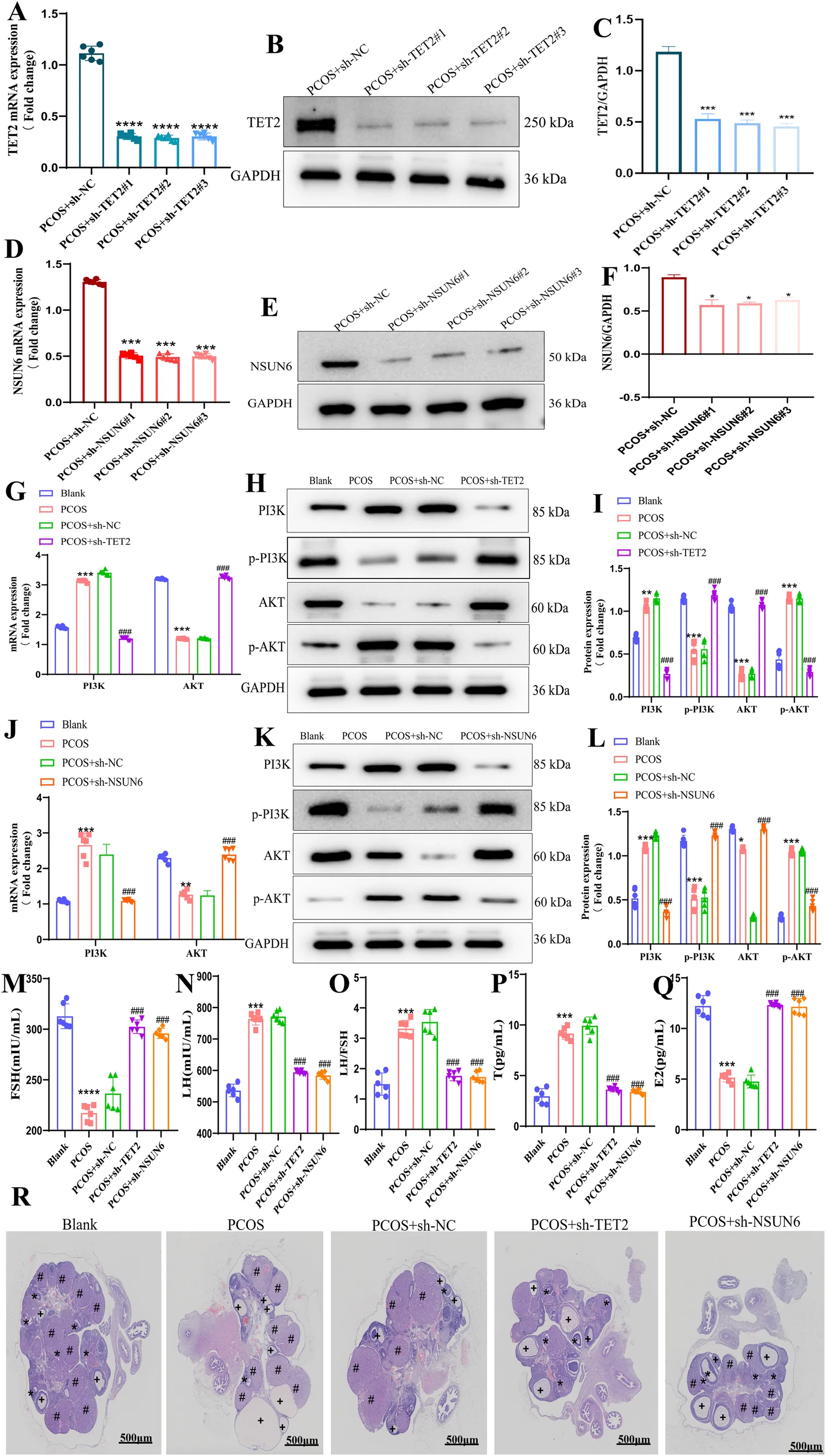
Effects of TET2 and NSUN6 knockdown on PI3K/AKT signaling, sex hormone levels, and ovarian morphology in DHEA-induced PCOS rats. (https://doi.org/10.6084/m9.figshare.33415486). (A–C) Knockdown efficiency of TET2 assessed by qRT-PCR and Western blotting. (D–F) Knockdown efficiency of NSUN6 assessed by qRT-PCR and Western blotting. (G–I) Effects of TET2 knockdown on PI3K and AKT mRNA expression and PI3K/AKT-related protein expression, including PI3K, p-PI3K, AKT, and p-AKT. (J–L) Effects of NSUN6 knockdown on PI3K and AKT mRNA expression and PI3K/AKT-related protein expression. (M–Q) Serum levels of follicle-stimulating hormone (FSH), luteinizing hormone (LH), the LH/FSH ratio, testosterone (T), and estradiol (E2), respectively, following TET2 or NSUN6 knockdown. (R) Representative hematoxylin and eosin-stained ovarian sections showing the effects of TET2 and NSUN6 knockdown on ovarian morphology.*: developing follicles; ^#^: corpus luteum; ^+^: cystic follicles. Data are presented as mean ± SEM; N = 6. Statistical significance was determined using Blank or PCOS+sh-NC. *P < 0.05, **P < 0.01, ***P < 0.001,****P < 0.0001. Statistical significance was determined using PCOS+sh-NC. ^#^*P* < 0.05, ^##^*P* < 0.01, ^###^*P* < 0.001,^####^*P* < 0.0001.

Subsequently, the effects of TET2 knock-down on the expression and phosphorylation status of PI3K/AKT signaling-related molecules were investigated. TET2 knock-down significantly altered the mRNA expression levels of PI3K and AKT, and affected the protein expression levels of PI3K, p-PI3K, AKT, and p-AKT (Fig 6G–6I). The ratios of p-PI3K/PI3K and p-AKT/AKT were further calculated to evaluate the relative phosphorylation status of PI3K and AKT. Compared with the PCOS model group and the PCOS+sh-NC group, TET2 knock-down reduced the p-PI3K/PI3K ratio, but increased the p-AKT/AKT ratio, suggesting that TET2 knock-down may exert differential regulatory effects on the phosphorylation of PI3K and AKT, leading to mismatch or remodeling of PI3K/AKT signaling rather than an overall attenuation of this pathway.

Similarly, NSUN6 knock-down also altered the expression of PI3K and AKT at both the mRNA and protein levels (Fig 6J–6L). Compared with the PCOS+sh-NC group, NSUN6 knock-down caused differential changes in the p-PI3K/PI3K and p-AKT/AKT ratios, indicating that NSUN6 may be involved in the phosphorylation regulation of PI3K/AKT signaling-related proteins and may lead to mismatch or remodeling of this signaling pathway.

We further assessed the effects of TET2 and NSUN6 knockdown on PCOS-associated endocrine abnormalities. Serum FSH, LH, the LH/FSH ratio, testosterone, and estradiol levels were measured (Fig 6M–6Q). Compared with the PCOS+sh-NC group, knockdown of TET2 or NSUN6 partially ameliorated the PCOS-associated abnormalities in serum FSH, LH, the LH/FSH ratio, testosterone, and estradiol levels. In addition, hematoxylin and eosin staining showed that TET2 or NSUN6 knockdown reduced cystic follicle formation and partially restored ovarian morphology in DHEA-induced PCOS rats (Fig 6R). Collectively, these findings suggest that TET2 and NSUN6 are functionally associated with PCOS-related endocrine and ovarian abnormalities and may contribute to the dysregulation of PI3K/AKT signaling.

## Discussion

Polycystic Ovary Syndrome (PCOS) exerts extensive adverse effects on women’s health and quality of life. Currently, the precise pathophysiology of PCOS remains incompletely elucidated, resulting in a lack of specific treatment plans [23,24]. Given the persistent increase in the occurrence rate of PCOS in recent years, coupled with a trend toward younger onset age, in-depth investigation of its pathophysiological mechanisms and the development of effective therapeutic interventions are of critical importance [25]. Aberrant DNA methylation and dysregulated epigenetic regulation of multiple genes have been observed in PCOS patients, collectively contributing to its pathological progression and the development of metabolic complications [5,26,27]. However, the precise methylation status of specific genes and the extent of methylation pattern disruption remain highly uncertain [28,29]. Given the reversible nature of epigenetic modifications, the identification of “druggable” regulatory regions capable of correcting abnormal gene expression holds potential therapeutic feasibility [30]. Therefore, research on DNA methylation in PCOS provides a promising avenue for the development of novel therapeutic strategies based on chromatin methylation mechanisms.

This study employed epitranscriptomic microarray technology to analyze the characteristics of m^5^C RNA modifications in a rat model of polycystic ovary syndrome (PCOS) induced by a hyperandrogenic environment. The results revealed increased m^5^C modification levels of TET2 and NSUN6 mRNAs in the ovarian tissues of PCOS rats, accompanied by elevated expression of their corresponding recognition proteins. A total of 1,090 RNAs exhibiting differential expression and methylation levels were identified. Bioinformatic analysis indicated that these differences were primarily associated with the Hippo signaling pathway, endocrine resistance mechanisms, thyroid hormone signaling pathway, cancer-related pathways, and cortisol synthesis pathways, which are closely related to metabolic abnormalities and insulin resistance [31].This study employed epitranscriptomic microarray technology to analyze the characteristics of m^5^C RNA modifications in a rat model of polycystic ovary syndrome (PCOS) induced by a hyperandrogenic environment. The results revealed increased m^5^C modification levels of TET2 and NSUN6 mRNAs in the ovarian tissues of PCOS rats, accompanied by elevated expression of their corresponding recognition proteins. A total of 1,090 RNAs exhibiting differential expression and methylation levels were identified. Bioinformatic analysis indicated that these differences were primarily associated with the Hippo signaling pathway, endocrine resistance mechanisms, thyroid hormone signaling pathway, cancer-related pathways, and cortisol synthesis pathways, which are closely related to metabolic abnormalities and insulin resistance [32–35].

Recent studies indicate that hyperactivation of the PI3K/AKT pathway can affect estrogen receptor (ER) function through multiple mechanisms [35–38]. AKT can directly phosphorylate ER, altering its transcriptional activity and subcellular localization, while concurrently activating downstream mTOR signaling to further modulate the expression of ER-related genes, thereby promoting the pathogenesis of polycystic ovary syndrome (PCOS) [39,40]. Furthermore, sustained activation of the PI3K/AKT pathway influences the phosphorylation of insulin receptor substrates, subsequently affecting glucose uptake and utilization, and contributing to the development of insulin resistance in PCOS [41,42]. In primordial follicles during the resting state, PTEN expression maintains the PI3K/AKT signaling at a low activity level [43]. Upon receiving activation signals, PI3K is activated, leading to subsequent AKT activation, which promotes the transition of primordial follicles to primary follicles [44]. This process involves the phosphorylation and inactivation of the transcription factor FoxO3a, thereby relieving its inhibitory effect on follicular activation [43,45]. Beyond the aforementioned functions, the PI3K/AKT pathway is also implicated in the inhibition of cell proliferation and the promotion of apoptosis, all of which may contribute to the pathogenesis and progression of PCOS [43,46]. Correspondingly, in our study, the expression of PI3K and AKT was detected via MeRIP-qPCR, Western blot, and immunohistochemistry, confirming the role of m^5^C methylation modification in the PI3K/AKT pathway in PCOS rats.

SRC (Src family tyrosine kinase), an upstream regulator of the PI3K-AKT pathway, also serves as a critical co-activator of the estrogen receptor (ER) [20]. SRC phosphorylates specific sites on ER, modifying its conformation and transcriptional activity, which can confer resistance to selective estrogen receptor modulators (such as tamoxifen) [47]. Concurrently, SRC family kinases can phosphorylate serine residues of insulin receptor substrate-1 (IRS-1); this phosphorylation inhibits tyrosine phosphorylation of IRS-1, thereby attenuating the efficiency of insulin signal transduction [48]. SRC can also modulate the expression of genes related to follicular activation by phosphorylating key signaling molecules, influencing the transition of follicles from a resting to an active state. As anticipated, the SRC gene was found to be significantly overexpressed in PCOS model rats in this study. Moreover, given the statistically significant difference in mRNA expression levels between the control and PCOS groups, SRC overexpression in PCOS model rats appears to be associated with higher m^5^C modification levels. Similarly, the iRNA-m^5^C program predominantly predicted m^5^C sites within the coding sequence of SRC mRNA in this study. Furthermore, investigations into the pathophysiology of PCOS have identified SRC as an important gene involved in lysosomal function and the regulation of ferroptosis, suggesting its potential as a therapeutic target for PCOS. However, the relationship between aberrant SRC expression and m^5^C methylation modification in PCOS remains unelucidated. Meanwhile, it has been reported that in endocrine resistance, activation of the SRC-PI3K/AKT axis provides robust survival signals for cancer cells [20,49]. m^5^C methylation of the SRC-PI3K/AKT axis may represent a key pathway underlying the development of phenotypes such as endocrine resistance and insulin resistance in PCOS.

Our KEGG enrichment analysis of the m^5^C methylation gene microarray also identified the thyroid hormone signaling pathway and pathways related to Cushing’s syndrome. In line with existing research findings, the thyroid hormone signaling pathway and Cushing’s syndrome pathway are involved in the pathogenesis of PCOS [50,51]. Notably, classical signaling pathways such as PI3K/AKT and MAPK can interact with the thyroid hormone signaling pathway and Cushing’s syndrome pathway through cross-regulation, thereby affecting the endocrine homeostasis and metabolic balance in PCOS [50,52]. Furthermore, as the core system regulating female reproductive endocrinology, the hypothalamic-pituitary-ovarian axis (HPOA) is also modulated by the thyroid hormone signaling pathway and Cushing’s syndrome, and plays a significant role in the development and progression of PCOS [10,18,53].

This study delineates the altered m5C methylation landscape in a PCOS rat model and suggests a potential regulatory link between m5C-related regulators, including NSUN6 and TET2, and the SRC/PI3K/AKT signaling pathway. Importantly, TET2 and NSUN6 knockdown was confirmed by qRT-PCR and Western blotting, and these interventions partially reversed the PCOS-associated abnormalities in PI3K/AKT-related protein expression, phosphorylation status, serum sex hormone levels, and ovarian morphology. These findings provide functional and phenotypic rescue evidence supporting the involvement of TET2 and NSUN6 in PCOS-related ovarian dysfunction.The opposite changes observed in the p-PI3K/PI3K and p-AKT/AKT ratios suggest differential regulation or uncoupling of PI3K and AKT signaling, rather than uniform activation or inhibition of the PI3K/AKT pathway. Therefore, our findings should be interpreted as evidence of PI3K/AKT signaling dysregulation rather than definitive evidence of pathway overactivation.

Several limitations should be considered. First, the DHEA-induced PCOS rat model primarily recapitulates androgen excess and related ovarian morphological abnormalities, but does not fully reproduce the complex and heterogeneous features of human PCOS. This model may not adequately reflect all metabolic abnormalities, insulin-resistance phenotypes, adiposity-related changes, or the diverse reproductive and endocrine manifestations observed in patients. Therefore, the present findings should be interpreted within the context of a hyperandrogenic PCOS model and should not be generalized to all PCOS phenotypes.Second, although our findings were validated ex vivo in primary granulosa cells, the use of a rat model and the absence of human granulosa cells or clinical samples limit the direct translation of these findings to human PCOS. Third, although MeRIP-qPCR and microarray analyses confirmed transcript-level m5C enrichment, these methods cannot resolve single-nucleotide methylation sites. Fourth, although TET2 and NSUN6 knockdown provided functional evidence and partially rescued PCOS-associated phenotypes, these experiments did not establish a direct causal link between specific m5C sites, TET2/NSUN6 activity, and PI3K/AKT signaling.

Future studies should validate these findings in additional PCOS models, human granulosa cells, and clinical cohorts; apply high-resolution approaches such as RNA bisulfite sequencing to map precise m5C loci; and perform complementary gain-of-function or pathway-specific epistasis experiments to further establish the underlying molecular mechanism. A complete mechanistic understanding of m5C regulation in PCOS is needed to assess its therapeutic potential. Final conclusions regarding the clinical relevance of these findings await further experimental confirmation.

## Conclusion

In conclusion, this study identified a significantly altered mRNA m5C methylation landscape in a DHEA-induced PCOS rat model and confirmed the upregulation of the m5C regulators NSUN6 and TET2 in primary ovarian granulosa cells. Knockdown of TET2 and NSUN6 partially rescued PCOS-associated abnormalities in PI3K/AKT-related signaling, serum sex hormone levels, and ovarian morphology. The opposite changes in the p-PI3K/PI3K and p-AKT/AKT ratios suggest differential or uncoupled regulation of PI3K/AKT signaling rather than uniform pathway activation or inhibition. These findings support a functional involvement of NSUN6 and TET2 in PCOS-associated ovarian dysfunction, but do not establish a direct causal link between specific m5C sites and pathway dysregulation. Because the DHEA model primarily mimics hyperandrogenism and MeRIP-based methods lack single-nucleotide resolution, further validation in human samples and high-resolution m5C mapping is required.

## Supporting information

S1 File The original plotting data, the original uncropped and unadjusted images underlying all blot or gel results for https://doi.org/10.6084/m9.figshare.33415252. (DOCX)

S1 Table Sequences of primers used for RT-qPCR analysis for https://doi.org/10.6084/m9.figshare.33415345. (DOCX)

S2 Table Prediction of SRC mRNA using the iRNA-m^5^C program identified four potential m^5^C sites with high confidence for https://doi.org/10.6084/m9.figshare.33415372. (XLSX)
