# Supplementary material for: Aberrant mRNA 5-methylcytosine modification contributes to ovarian dysfunction in a rat model of polycystic ovary syndrome
Source: PLoS One. 2026 Oct 27;21(10):e0358238. doi: 10.1371/journal.pone.0358238 (PMC13712706; doi:10.1371/journal.pone.0358238)
Supplement: Supplementary file 1 [file pone.0358238.s001.docx]

**Fig.S4 Src_Rawblot**

Src(60kDa)

Fig. S4 Original Western blot of Src.

The uncropped Western blot image shows Src protein (~60 kDa) levels in each experimental group as reported in the main figures.

**Fig.S4 PI3K_Rawblot.**

PI3K (85kDa)

Fig. S4 Original Western blot of PI3K.

The raw blot image of the loading PI3K (~85 kDa) is provided to support the accuracy of protein quantification.

**Fig.S4 AKT _Rawblot.**

AKT(60 kDa)

Fig. S4 Original Western blot of AKT (independent replicate).

This raw, unedited image from an independent experiment shows AKT (~60 kDa) expression, supporting the reproducibility of the reported findings.

**Fig.S4 GAPDH _Rawblot.**

GAPDH（36kDa）

Fig. S4 Original Western blot of GAPDH (independent replicate).

The full-length raw blot of GAPDH (~36kDa) was obtained from a separate experiment, confirming the consistency of the results.

**Fig.S5 TET2_Rawblot**

TET2 (250kDa)

Fig. S5 Original Western blot of TET2 (independent replicate).

This uncropped blot image shows TET2 (~250 kDa) bands from an independent repetition, further validating the expression analysis.

**Fig.S5 GAPDH _Rawblot.**

GAPDH（36kDa）

Fig. S5 Original Western blot of GAPDH (independent replicate).

The full-length raw blot of GAPDH (~36kDa) was obtained from a separate experiment, confirming the consistency of the results.

**Fig.S5 NSUN6_Rawblot**

NSUN6(26kDa)

Fig. S5 Original Western blot of NSUN6 (independent replicate).

This uncropped blot image shows NSUN6 (~50 kDa) bands from an independent repetition, further validating the expression analysis.

**Fig.S5 GAPDH _Rawblot.**

GAPDH（36kDa）

Fig. S5 Original Western blot of GAPDH (independent replicate).

The full-length raw blot of GAPDH (~36kDa) was obtained from a separate experiment, confirming the consistency of the results.

**Fig.S5 PI3K_Rawblot**

PI3K (85kDa)

Fig. S5 Original Western blot of PI3K (independent replicate).

This uncropped blot image shows PI3K (~85 kDa) bands from an independent repetition, further validating the expression analysis.

**Fig.S5 p-PI3K_Rawblot**

p-PI3K (85kDa)

Fig. S5 Original Western blot of PI3K (independent replicate).

This uncropped blot image shows p-PI3K (~85 kDa) bands from an independent repetition, further validating the expression analysis.

**Fig.S5 AKT_Rawblot**

AKT(60kDa)

Fig. S5 Original Western blot of AKT(independent replicate).

This uncropped blot image shows AKT (~60 kDa) bands from an independent repetition, further validating the expression analysis.

**Fig.S5 p-AKT_Rawblot**

AKT(60kDa)

Fig. S5 Original Western blot of p-AKT(independent replicate).

This uncropped blot image shows p-AKT (~60 kDa) bands from an independent repetition, further validating the expression analysis.

**Fig.S5 GAPDH_Rawblot**

GAPDH(36kDa)

Fig. S5 Original Western blot of GAPDH (independent replicate).

This uncropped blot image shows GAPDH(~36kDa) bands from an independent repetition, further validating the expression analysis.

**Fig.S5 PI3K_Rawblot**

PI3K (85kDa)

Fig. S5 Original Western blot of PI3K (independent replicate).

This uncropped blot image shows PI3K (~85 kDa) bands from an independent repetition, further validating the expression analysis.

**Fig.S5 p-PI3K_Rawblot**

p-PI3K (85kDa)

Fig. S5 Original Western blot of PI3K (independent replicate).

This uncropped blot image shows p-PI3K (~85 kDa) bands from an independent repetition, further validating the expression analysis.

**Fig.S5 AKT_Rawblot**

AKT(60kDa)

Fig. S5 Original Western blot of AKT(independent replicate).

This uncropped blot image shows AKT (~60 kDa) bands from an independent repetition, further validating the expression analysis.

**Fig.S5 p-AKT_Rawblot**

AKT(60kDa)

Fig. S5Original Western blot of p-AKT(independent replicate).

This uncropped blot image shows p-AKT (~60 kDa) bands from an independent repetition, further validating the expression analysis.

**Fig.S5 GAPDH_Rawblot**

GAPDH(36kDa)

Fig. S5| Original Western blot of GAPDH (independent replicate).

This uncropped blot image shows GAPDH(~36kDa) bands from an independent repetition, further validating the expression analysis.
