# Supplementary material for: Aberrant mRNA 5-methylcytosine modification contributes to ovarian dysfunction in a rat model of polycystic ovary syndrome
Source: PLoS One. 2026 Oct 27;21(10):e0358238. doi: 10.1371/journal.pone.0358238 (PMC13712706; doi:10.1371/journal.pone.0358238)
Supplement: Supplementary file 2 [file pone.0358238.s002.docx]

**Table S1 Sequences of primers used for RT-qPCR analysis**

| Locus_tag | Forward primer (5′-3′) | Reverse primer (5′-3′) |
| --- | --- | --- |
| TET2 | AATCGCCTTCGGATTCAGACACTC | CTTGACCTCCGATACACCCATTTAGC |
| NSUN6 | AGCAGTGGAGAGCAGAGGAA | TCTGGTGGTGATGATGGTGA |
| SRC | TGCTGTACAAGTCCATCCAG | CTTGTAGATCTTGGGCACCT |
| PI3K | TGTGAACGACTATCAGCAGG | CACTGTACTCCACGAACATC |
| AKT | GTCATCGAACGCACCTTCCAT | AGCTTCAGGTACTCAAACTCGT |
| β-Actin | CCTCCGCCCTCTCTCATTTA | AGTGTTGTGCAGTTGTCTCG |

**Table S2 Sequences of primers used for MeRIP-qPCR analysis**

| Locus_tag | Forward primer (5′-3′) | Reverse primer (5′-3′) |
| --- | --- | --- |
| TET2 | GAGGCTGTGGAGTTTGAGGA | GAGGCTGTGGAGTTTGAGGA |
| NSUN6 | TGCTGTGAGTACCTGCTGGT | GGTGATGGTGAGGATGAGGA |
| SRC | CTGGTGTTCTGCTGCTGTTC | AGGTCTTCCTGGTGGTGATG |
| PI3K | TGGACCATCCACGACATCTA | CTGGTGATGATGGTGGTGTT |
| AKT | AGCGACGTGGCTATTGTGAA | CTGGCTGAGTAGGAGAACTGG |
| β-Actin | GGAGATTACTGCCCTGGCTCCTAGC | GGCCGGACTCATCGTACTCCTGCT |
